# Effects of Norspermidine on Dual‐Species Biofilms Composed of *Streptococcus mutans* and *Streptococcus sanguinis*

**DOI:** 10.1155/2019/1950790

**Published:** 2019-11-03

**Authors:** Yan Sun, Yihuai Pan, Yu Sun, Mingyun Li, Shengbin Huang, Wei Qiu, Huanxin Tu, Keke Zhang

**Affiliations:** ^1^Department of Endodontics, School and Hospital of Stomatology, Wenzhou Medical University, Wenzhou, China; ^2^State Key Laboratory of Oral Diseases, West China Hospital of Stomatology, Sichuan University, Chengdu, Sichuan, China; ^3^Institute of Stomatology, School and Hospital of Stomatology, Wenzhou Medical University, Wenzhou, China; ^4^School and Hospital of Stomatology, Wenzhou Medical University, Wenzhou, China

## Abstract

The present study aimed at investigating the influence of norspermidine on the formation of dual-species biofilms composed of *Streptococcus mutans* (*S. mutans*) and *Streptococcus sanguinis* (*S. sanguinis*). Crystal violet assay was conducted to assess the formation of single-species biofilms of *S. mutans* and *S. sanguinis*, and the growth curve was carefully observed to monitor the growth of these two species of bacteria. Fluorescence in situ hybridization (FISH) and MTT array were used to analyze the composition and metabolic activity of the dual-species biofilms, respectively. Extracellular polysaccharides (EPS)/bacteria staining, anthrone method, and scanning electron microscopy (SEM) imaging were conducted to study the synthesis of EPS by dual-species biofilms. Lactic acid assay and pH were measured to detect dual-species biofilm acid production. We found that norspermidine had different effects on *S. mutans* and *S. sanguinis* including their growth and biofilm formation. Norspermidine regulated the composition of the dual-species biofilms, decreased the ratio of *S. mutans* in dual-species biofilms, and reduced the metabolic activity, EPS synthesis, and acid production of dual-species biofilms. Norspermidine regulated dual-species biofilms in an ecological way, suggesting that it may be a potent reagent for controlling dental biofilms and managing dental caries.

## 1. Introduction

The diverse community of microorganisms embedded in extracellular polymeric substances on the tooth surface is known as dental plaque [1]. According to the ecological plaque hypothesis, an adverse ecological shift in the equilibrium of the oral microbiota, driven by changes in the dental environment, will lead to dental caries [2]. When the dental biofilm is frequently exposed to dietary carbohydrates, the composition of dental biofilms shifts to cariogenic bacteria such as *Streptococcus mutans* (*S. mutans*), lactobacilli, while the beneficial bacteria such as *Streptococcus sanguinis* (*S. sanguinis*) was inhibited [2, 3]. *S. mutans* plays a critical role in the development of dental caries due to its biofilm formation, extracellular polysaccharide (EPS) production, acidogenicity, and acidurity. [4, 5]. *S. sanguinis* is a pioneer colonizer of oral biofilms, and its presence is considered relevant to the absence of dental caries [6]. The relationship between *S. mutans* and *S. sanguinis* has been reported as competitive and antagonistic, with high levels of one bacterium correlated with low levels of the other [7]. Additional studies revealed that they compete with each other by producing hydrogen peroxide (*S. sanguinis*) and mutacin (*S. mutans*) [8]. However, this relationship between the two strains could simply be an artifact of more complicated interactions in the dental biofilm [9].

Norspermidine, a kind of polyamines, has been reported as essential for biofilm formation in *Vibrio cholerae* [10]. In 2012, Ilana Kolodkin-Gal et al. [11] found that *Bacillus subtilis* (*B. subtilis*) produces norspermidine to induce biofilm disassembly during the biofilm life cycle [11]. In addition, this biofilm disassembly factor could inhibit the biofilm formation of *B. subtilis*, *Staphylococcus aureus* (*S. aureus*), and *Escherichia coli* (*E. coli*). However, some of the results reported by Ilana Kolodkin-Gal et al. were challenged by a subsequent research in which *B. subtilis* was lacking the biosynthetic pathway for norspermidine, and the concentration of norspermidine required to inhibit *B. subtilis* biofilm formation was much higher [11]. Nonetheless, several studies in recent years have explored the effect of norspermidine on other single-species bacterial biofilms such as *Staphylococcus epidermidis* (*S. epidermidis*), *Salmonella enterica* (*S. enterica*), *Pseudomonas aeruginosa* (*P. aeruginosa*), *Acinetobacter baumannii* (*A. baumannii*), *Klebsiella pneumoniae* (*K. pneumonia*), and *S. mutans*. These results have shown that various species have dissimilar sensitivities to norspermidine [12–16]. Norspermidine induced the suppression and disassembly of biofilm formation of clinical or commensal strains of *S. epidermidis*, except one strain [12]. Moreover, norspermidine could not only inhibit biofilm formation but also eradicate mature biofilms in *P. aeruginosa* [13]. In contrast, in *S. enterica*, norspermidine was not a potential inhibitor of biofilm [14]. Significantly, norspermidine displayed the ability to inhibit and disperse biofilms in multidrug-resistant clinical isolates related to persistent infections in wounds to extremities [15].

The effect of norspermidine on the *S. mutans* biofilm has attracted more attention lately, and results have revealed that norspermidine inhibits the biofilm formation of *S. mutans*, while also changing the basic structure of the biofilm [16]. Nevertheless, there is no evidence on the effects of norspermidine on dual-species biofilms composed of *S. mutans* and *S. sanguinis*. Thus, we conducted the present study to investigate the influence of norspermidine on the composition and cariogenic virulence of dual-species biofilms.

## 2. Materials and Methods

### 2.1. Bacterial Strains and Growth Conditions

*S. mutans* UA159 and *S. sanguinis* ATCC 10556 were routinely grown in Brain Heart Infusion Broth (BHI, Oxoid, Basingstoke, UK) at 37°C under 5% CO_2_ (v/v). For generating the growth curve, BHI was used as the culture media. For biofilm formation, this media was supplemented with 1% sucrose (m/v).

### 2.2. Biofilm Formation

For formation of a single-species biofilm, an overnight culture of bacteria was placed in a 96-well microtiter plate at a final concentration of 10^6^ CFU/mL, together with different concentrations of norspermidine, for 24 h (final volume of 200 *μ*L). For formation of dual-species biofilms, sterile glass slides were placed into a 24-well plate, two single species were cultured overnight and then combined into a culture cluster at a final concentration of 10^6^ CFU/mL, together with different concentrations of norspermidine (final volume of 2 mL), and incubated for 24 h. Hydrochloric acid was used to adjust the media pH. A group cultured in the absence of norspermidine (0.0 mM) was used as a control.

### 2.3. Crystal Violet Assay

Crystal violet assay was used to assess biofilm formation and conducted as previously described [17]. For crystal violet staining, single-species biofilms in 96-well plates were fixed with methanol for 15 min. After the supernatant was discarded, the plate was air-dried, and 100 *μ*L of 0.1% (w/v) crystal violet was added into each well and incubated for 20 min. The stained biofilms were then visualized using a stereo microscope (Nikon SMZ800, Nikon Corporation, Japan). For quantitative analysis, the bound crystal violet was dissolved in 33% acetic acid and read at an absorbance of 590 nm by a microplate reader (SpectraMax M5, Molecular Devices, USA).

### 2.4. Fluorescence In Situ Hybridization (FISH)

FISH was used to analyze the composition of dual-species biofilms as previously described [18]. Briefly, biofilms were washed twice with PBS and fixed with 4% paraformaldehyde for 12 h. After the bacterial cell walls were partially lysed by lysozyme, the biofilms were incubated using an ethanol gradient (50%, 80%, and 96%) for 3 min at each concentration. The biofilms were dried at 46°C for 10 min followed by hybridization with species-specific probes (Supplementary Appendix [Supplementary-material supplementary-material-1]). The biofilms were observed by a Nikon confocal laser scanning microscope (CLSM, Nikon A1, Nikon Corporation, Japan). Five random areas from each sample were captured for semiquantitative analysis. The *S. sanguinis* ratio was analyzed with Image-Pro Plus 6.0 (Media Cybernetics, Inc., Silver Spring, MD, USA) according to the area of bacterial coverage.

### 2.5. Growth Curve

To monitor the effects of norspermidine on bacterial growth, growth curve was generated as previously described [19]. Bacteria cultured overnight were added to a 96-well microtiter plate at a final concentration of 10^6^ CFU/ml, together with different concentrations of norspermidine for 24 h (final volume of 200 *μ*L). The optical density (OD) at 600 nm was measured every 1 h with a microplate reader (SpectraMax M5, Molecular Devices, USA).

### 2.6. MTT Assay

MTT assay for dual-species biofilms was performed as previously described [20]. Following 24 h, biofilms were washed twice with PBS and moved to a new 24-well plate. Next, 0.5 mg/mL MTT was added to each well (1 mL) and incubated for 1 h at 37°C in 5% CO_2_. After 1 h, the biofilms were transferred to a new 24-well plate, and 1 mL of dimethyl sulfoxide (DMSO) was added to each well and incubated for 20 min. After pipette mixing, 200 *μ*l of solution was used to read the OD at 540 nm by a microplate reader (SpectraMax M5, Molecular Devices, USA).

### 2.7. Lactic Acid and pH Measurement

Lactic acid and pH measurement were conducted to monitor acid production [21]. After 24 h, dual-species biofilms were washed with cysteine peptone water (CPW) and then transferred to a new 24-well plate. After 1.5 ml of buffered peptone water (BPW) containing 0.2% sucrose was added to each well, the new plate was incubated at 5% CO_2_, 37°C for 3 h to allow acid production. Lactate dehydrogenase was used to quantify the lactate concentrations in the BPW solutions. The absorbance was monitored at 340 nm, and standard curves were generated using a lactic acid standard (Supplementary Appendix [Supplementary-material supplementary-material-1]). The pH of the supernatant before and after the 24 h formation of dual-species biofilms was measured by a pH meter (Mettler Toledo Instruments Co. Ltd., Shanghai, China).

### 2.8. Scanning Electron Microscopy (SEM) Imaging

To observe the morphology and EPS of dual-species biofilms, SEM imaging was performed [22]. After 24 h, dual-species biofilms were fixed with 2.5% glutaraldehyde and were dehydrated using an ethanol gradient. The biofilms were then sputter-coated with gold for observation by SEM (Quanta 200, FEI, Hillsboro, OR, USA).

### 2.9. Bacterial/Extracellular Polysaccharide Staining

Bacterial/extracellular polysaccharide (EPS) staining was carried out as previously described [23]. In brief, 2.5 *μ*M Alexa Fluor 647-dextran conjugate (Molecular Probes, Invitrogen Corp., Carlsbad, CA, USA) was added to each well at the beginning of biofilm formation. After 24 h, the bacteria were stained with 2.5 *μ*M SYTO 9 (Molecular Probes, Invitrogen Corp., Carlsbad, CA, USA). Next, CLSM was used to observe the biofilms. The bacteria were stained green by SYTO 9 (excitation/emission maxima are 480 nm/500 nm) while the polysaccharides were stained red by Alexa Fluor 647-dextran conjugate (excitation/emission maxima are 650 nm/668 nm). Next, the biofilms were evaluated with a CLSM (Nikon A1, Nikon Corporation, Japan). Five random pictures of each sample were acquired for semiquantitative analysis, and the EPS/bacteria ratio was analyzed with Image-Pro Plus 6.0 (Media Cybernetics, Inc., Silver Spring, MD, USA) according to the coverage area.

### 2.10. Water-Insoluble Exopolysaccharide Measurement

The water-insoluble exopolysaccharides of dual-species biofilms were measured by the anthrone method [24]. Briefly, the biofilms were collected and washed twice with sterile water and then resuspended in 0.4 M NaOH. After centrifugation, 300 *μ*L supernatant and 600 *μ*l anthrone reagent were mixed and incubated at 95°C for 6 min. The absorbance was read at OD 625 nm with microplate reader (SpectraMax M5, Molecular Devices, USA). Standard curves were prepared with a dextran standard (Supplementary Appendix [Supplementary-material supplementary-material-1]).

### 2.11. Data Analysis

One-way analysis of variance (ANOVA) was performed to evaluate the significance between the variables, followed by Tukey's multiple comparison test. A *p* value ≤0.05 was considered statistically significant. All statistical analyses were performed using the SPSS software 16.0 (SPSS Inc., Chicago, IL, USA).

## 3. Results

### 3.1. Biofilm Formation of *S. mutans* and *S. sanguinis* Showed Differing Sensitivities to Norspermidine

**Figure 1:**
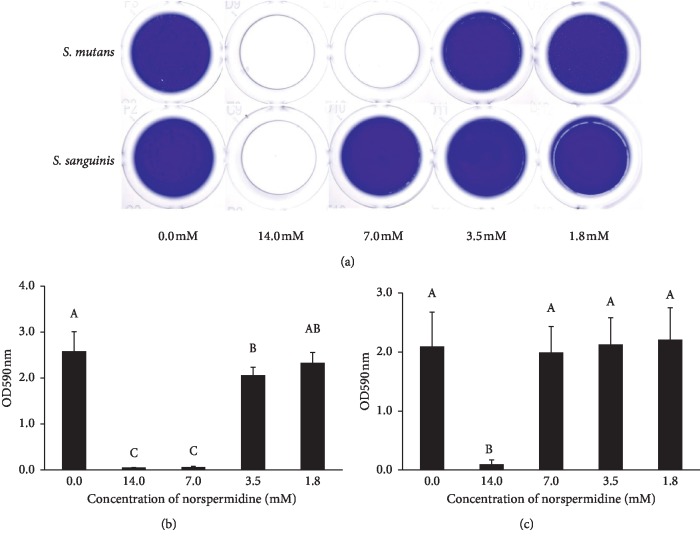
Single-species biofilm formation under the effects of norspermidine: (a) crystal violet staining of *S. mutans* and *S. sanguinis* biofilms under the effects of norspermidine; quantitative analysis of crystal violet staining of *S. mutans* biofilm (b) and *S. sanguinis* biofilm (c) under the effects of norspermidine. Data are presented as mean ± standard deviation, and values with dissimilar letters are significantly different from each other (*p* < 0.05).

The crystal violet staining results showed that *S. mutans* and *S. sanguinis* could barely form biofilms in the presence of 7.0 mM and 14.0 mM norspermidine (Figure 1(a)). The quantitative results of crystal violet assay showed a similar trend, with norspermidine leading to 20.4% (concentration at 3.5 mM, *p* < 0.05) and 9.8% (concentration at 1.8 mM, *p* > 0.05) reductions in OD value of *S. mutans* compared with the control (Figure 1(b)). Contrastingly, in *S. sanguinis*, there were no significant differences in OD value in the presence of 7.0 mM, 3.5 mM, and 1.8 mM norspermidine when compared with the control (*p* > 0.05; Figure 1(c)). Collectively, these results suggest that norspermidine exerted an antibiofilm effect in a strain-dependent manner.

### 3.2. Norspermidine Altered the Composition of *S. mutans* and *S. sanguinis* Dual-Species Biofilms

**Figure 2:**
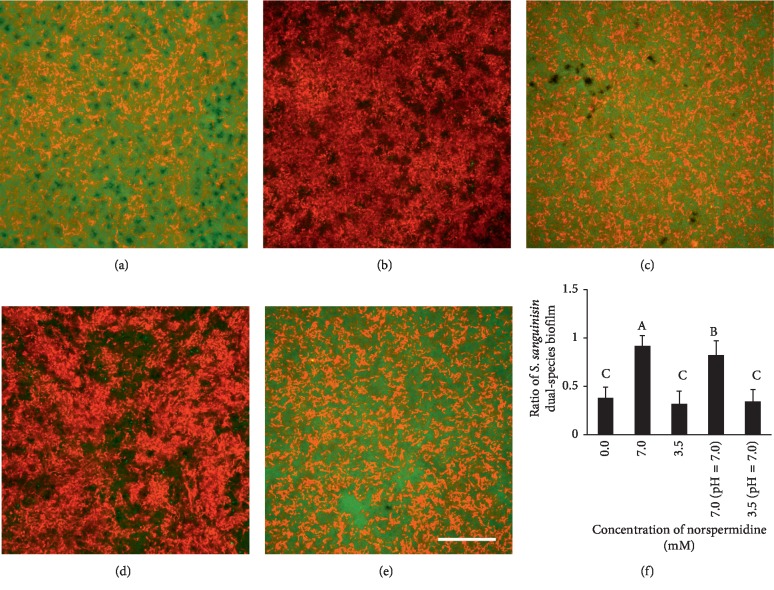
Composition of *S. mutans* and *S. sanguinis* dual-species biofilms as revealed by fluorescence in situ hybridization (FISH): (a)–(e) Dual-species biofilms in the 0.0 mM norspermidine group, 7.0 mM norspermidine group, 3.5 mM norspermidine group, pH-adjusted (pH = 7.0) 7.0 mM norspermidine group, and pH-adjusted (pH = 7.0) 3.5 mM norspermidine group, respectively (*S. mutans* was stained green and *S. sanguinis* was stained red, scale bar = 50 *μ*m); (f) *S. sanguinis* ratio in dual-species biofilms according to coverage area. Data are presented as mean ± standard deviation, and values with dissimilar letters are significantly different from each other (*p* < 0.05).

The FISH results demonstrated that *S. sanguinis* displayed a significant competitive advantage over *S. mutans* in the 7.0 mM and pH-adjusted (pH = 7.0) 7.0 mM norspermidine groups, when compared with the other groups (Figures 2(a)–2(e)). According to the semiquantitative results, regardless of the pH adjustment of the culture media to 7.0, the 3.5 mM norspermidine groups produced no significant effect on the composition of the dual-species biofilms when compared with the control (*p* > 0.05; Figure 2(f)). However, the *S. sanguinis* ratio was much higher in the 7.0 mM norspermidine groups (*p* < 0.05; Figure 2(f)), reaching 91% ± 10% in the 7.0 mM norspermidine group and 85% ± 15% in the pH-adjusted (pH = 7.0) 7.0 mM norspermidine group. According to these results, norspermidine seemed to be able to regulate the dual-species biofilm and gave *S. sanguinis* a competitive edge over *S. mutans*.

### 3.3. Norspermidine Decreased the EPS Synthesis of Bacteria within Biofilms

**Figure 3:**
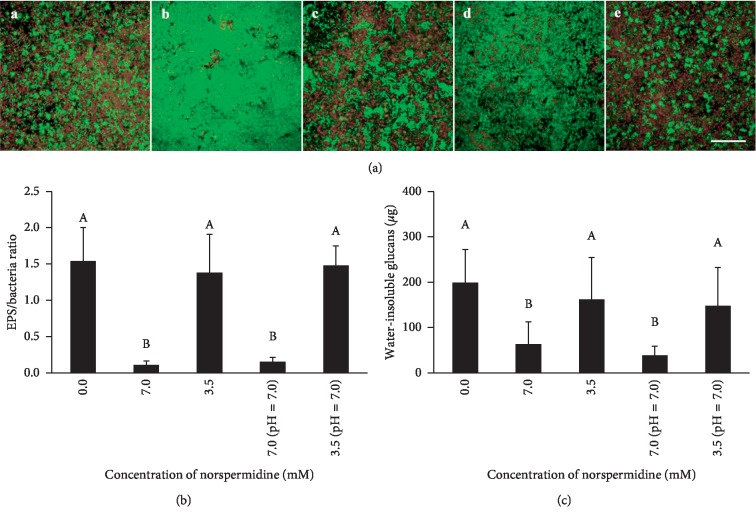
Extracellular polysaccharides (EPS) in *S. mutans* and *S. sanguinis* dual-species biofilms. (a) EPS and bacterial staining of dual-species biofilm: (A–E) biofilm staining in the 0.0 mM norspermidine group, 7.0 mM norspermidine group, 3.5 mM norspermidine group, pH-adjusted (pH = 7.0) 7.0 mM norspermidine group, and pH-adjusted (pH = 7.0) 3.5 mM norspermidine group, respectively (bacteria were stained green and EPS was stained red, scale bar = 50 *μ*m); (b) EPS/bacteria ratio in dual-species biofilm according to coverage area; (c) water-insoluble glucans in biofilm of different groups. Data are presented as mean ± standard deviation, and values with dissimilar letters are significantly different from each other (*p* < 0.05).

**Figure 4:**
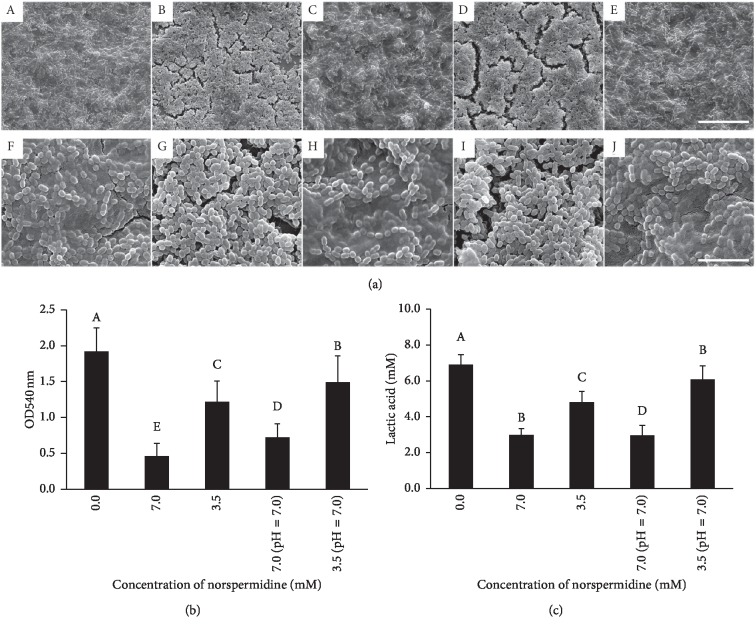
SEM imaging, metabolic activity, and acid production of *S. mutans* and *S. sanguinis* dual-species biofilms. (a) SEM imaging of dual-species biofilms: (A–E) biofilm in the 0.0 mM norspermidine group, 7.0 mM norspermidine group, 3.5 mM norspermidine group, pH-adjusted (pH = 7.0) 7.0 mM norspermidine group, and pH-adjusted (pH = 7.0) 3.5 mM norspermidine group, respectively (5000x, scale bar = 20 *μ*m); (F–J) biofilm in the 0.0 mM norspermidine group, 7.0 mM norspermidine group, 3.5 mM norspermidine group, pH-adjusted (pH = 7.0) 7.0 mM norspermidine group, pH-adjusted (pH = 7.0) 3.5 mM norspermidine group, respectively (20000x, scale bar = 5 *μ*m); (b) metabolic activity of dual-species biofilm in different groups as revealed by MTT assay; (c) lactic acid production of dual-species biofilm revealed by lactic acid measurement. Data are presented as mean ± standard deviation, and values with dissimilar letters are significantly different from each other (*p* < 0.05).

The bacterial/extracellular polysaccharides staining as observed by CLSM revealed that whether or not the media pH was adjusted to 7.0, norspermidine reduced EPS synthesis in the 7.0 mM groups (Figure 3(a)). There were higher EPS/bacteria ratios in the control, 3.5 mM norspermidine, and pH-adjusted 3.5 mM norspermidine groups, as compared to the 7.0 mM norspermidine and pH-adjusted 7.0 mM norspermidine groups (*p* < 0.05; Figure 3(b)). Likewise, the water-insoluble glucans produced by dual-species biofilms in the 7.0 mM norspermidine and pH-adjusted 7.0 mM norspermidine groups were also significantly reduced (*p* < 0.05; Figure 3(c)). Furthermore, the SEM images of dual-species biofilms confirmed that there were significantly fewer EPS in the 7.0 mM norspermidine and pH-adjusted 7.0 mM norspermidine groups when compared with the other three groups, all of which were tightly enmeshed in EPS (Figure 4(a)).

### 3.4. Norspermidine Reduced the Metabolic Activity of the Dual-Species Biofilms

An MTT assay was conducted to determine the effects of norspermidine on the metabolic activity of dual-species biofilms. The MTT results verified that, regardless of adjustment of the media pH to 7.0, both 7.0 mM and 3.5 mM norspermidine groups displayed reduced OD values (*p* < 0.05; Figure 4(b)). In particular, the 7.0 mM and pH-adjusted 7.0 mM (pH = 7.0) norspermidine groups displayed OD values decreased by around 75.9% and 62.4%, respectively. Based on the MTT results, norspermidine reduced the metabolic activity of dual-species biofilms composed of *S. mutans* and *S. sanguinis*.

### 3.5. Norspermidine Abrogated the Acid Production of Dual-Species Biofilms

Regardless of the pH adjustment of the norspermidine groups to 7.0, the pH of the supernatant after 24 h of dual-species biofilm formation was not changed significantly. The pH values were as follows: ∼4.20 (control group), 4.54 (7.0 mM norspermidine group, 8.30 pH at the beginning), 4.39 (3.5 mM norspermidine group, 7.77 pH at the beginning), 4.47 (pH-adjusted (pH = 7.0) 7.0 mM norspermidine group), and 4.27 (pH-adjusted (pH = 7.0) 3.5 mM norspermidine group). However, lactic acid production by the biofilm was significantly reduced in the groups containing norspermidine (Figure 4(b)). Notably, the 7.0 mM and pH-adjusted (pH = 7.0) 7.0 mM norspermidine groups were reduced to about 48.7% and 48.9% of control, respectively (*p* < 0.05).

### 3.6. Norspermidine Had Differing Effects on the Growth of *S. mutans* and *S. sanguinis*

**Figure 5:**
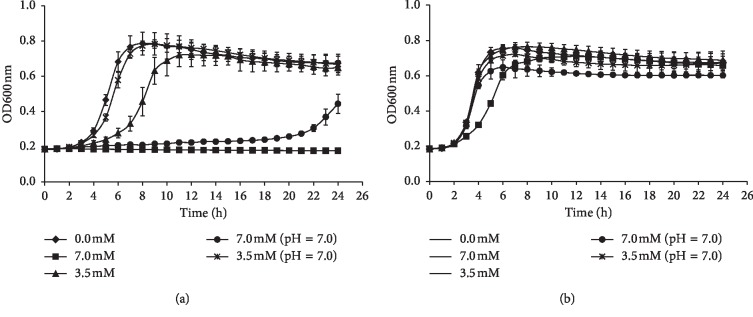
Growth curves of (a) *S. mutans* and (b) *S. sanguinis* at different concentrations of norspermidine. Data are presented as mean ± standard deviation.

To monitor the effects of norspermidine on the growth of *S. mutans* and *S. sanguinis*, a 24 h growth curve was generated. Based on the growth curve, we found that different concentrations of norspermidine at different pH values had little effect on the growth of *S. sanguinis* (Figure 5(b)). In contrast, for *S. mutans*, 7.0 mM norspermidine had a significant inhibitory effect on growth, and this inhibitory action could benefit from an alkaline pH (Figure 5(a)). These results suggest that the growth of *S. mutans* is more sensitive to norspermidine, compared to that of *S. sanguinis*.

## 4. Discussion

In the present study, we investigated the effects of norspermidine on the composition and cariogenic virulence of dual-species biofilms composed of *S. mutans* and *S. sanguinis*. The results showed that norspermidine had different effects on *S. mutans* and *S. sanguinis*, including on their growth and biofilm formation. Norspermidine not only regulated the composition of dual-species biofilms by decreasing the ratio of *S. mutans* but also reduced the metabolic activity, EPS synthesis, and acid production of dual-species biofilms. *S. mutans* and *S. sanguinis* exhibited different sensitivities to norspermidine, consistent with previous reports in which norspermidine had an inhibitory effect on biofilm formation dependent on species [14, 15]. The difference in antibiofilm activities of norspermidine seen in the present study might largely result from different suppression effect of norspermidine on bacterial growth. In addition, the dissimilar antibiofilm activities might be related to species differences in biofilm development, EPS composition, or other mechanisms hindering biofilm formation, as reported previously [15].

The composition of dual-species biofilms in the present study was altered, and *S. sanguinis* achieved a competitive advantage over *S. mutans* under the effects of 7.0 mM norspermidine. This result was probably due to norspermidine reacting differently to *S. mutan*s and *S. sanguinis* biofilm formation, with *S. mutan*s biofilm formation being more sensitive to norspermidine than *S. sanguinis*. Dental plaque is a complex microbial biofilm, and its balance is of great importance to stay healthy [25]. To control dental caries in an ecological way, drugs should specifically target the cariogenic bacteria (such as *S. mutans*) without influencing the rest of the oral biota or inhibit virulence (such as glucan and acid production) without affecting microbial viability [26]. In addition, enhancing the growth of health-associated bacteria (such as *S. sanguinis*) may be beneficial for rebalancing the ecology of the biofilm [26]. Furthermore, from a clinical point of view, enhancing the advantages of *S. sanguinis* over *S. mutans* is relevant to caries prevention [9]. From this perspective, norspermidine may be beneficial in reducing dental caries.

EPS plays an important role in the pathogenesis of dental caries through contributing to microbial binding, providing a cohesive diffusion-limiting 3D scaffold, providing fermentable sugars, and promoting the formation of acidic environments [27, 28]. The decreased EPS in the dual-species biofilm in the present study could be attributed to several aspects. Firstly, though the effect of norspermidine on EPS production by *S. sanguinis* was unknown, norspermidine downregulated the gene expression of *gtfB*, *gtfC*, and *gtfD*, which encode glucosyltransferases (Gtfs) for the production of water-insoluble glucans, water-soluble and water-insoluble glucans, and water-soluble glucans, respectively, from sucrose in *S. mutans* [16]. Glucans (mainly water-insoluble glucans) are recognized as key components of EPS in cariogenic biofilms [29]. Consequently, norspermidine reduced the EPS production of *S. mutans* and further diminished the total EPS of this dual-species biofilm. Secondly, since *S. mutans* and *S. sanguinis* react differently to norspermidine, it altered the composition of dual-species biofilms and reduced the ratio of *S. mutans*. *S. sanguinis* harbors only one glucosyltransferase, the GtfP, which is encoded by the gene *gtfP*, and is responsible for the synthesis of water-soluble glucans [30]. *S. mutans* was the main producer of insoluble glucans among oral bacteria [29]. According to a previous study, *S. mutans* produced much more EPS than *S. sanguinis* visually [6]. Therefore, the altered composition caused by norspermidine was also responsible for the reduction of EPS within the dual-species biofilm. Furthermore, the reduced metabolic activity of the biofilm may also contribute to the decreased total EPS.

Sucrose, a kind of disaccharide, can be directly broken down to form fructose and glucose. It is widely known as the most cariogenic sugar, since it is a substrate for the synthesis of polysaccharides and can be utilized by bacteria to produce large amounts of organic acids quickly [31–33]. The low pH induced by sucrose fermentation further promotes tooth decay. The lower ratio of *S. mutans* in the dual-species biofilm and lower metabolic activity of the biofilm might lead to decreased lactic acid production in the norspermidine-containing groups. Lactic acid is produced by *S. mutans* as the major end-product of glycolysis, in the presence of excess glucose [34]. Although *S. sanguinis* can also metabolize glucose to produce lactate, *S. mutans* produces more acid than *S. sanguinis* due to its higher ATP-glucose phosphotransferase activity [35]. Moreover, interestingly, norspermidine raised the pH of the culture media, which could benefit the control of dental caries.

According to previous studies, the mechanisms of inhibiting biofilm formation and disassembly by norspermidine are different. For instance, for biofilm dispersal, norspermidine interacted directly and specifically with the exopolysaccharide in *B. subtilis*, multispecies wastewater biofilms [11, 36]. But in *A. baumannii*, the inhibitory effects of norspermidine on biofilm formation might be due to inhibition of bacterial motility and quorum sensing (QS), both of which are essential to its biofilm formation [15]. A separate study indicated that norspermidine inhibited the swimming motility phenotype of *P. aeruginosa*, and QS-related gene expression was downregulated when norspermidine was cocultured with planktonic bacteria [13]. In *S. mutans*, the QS system was influenced by norspermidine during biofilm formation and consequently led to alterations in the biofilm structure. Based on the current research, since QS systems are often affected during biofilm formation, we hypothesized that the antibiofilm effects of norspermidine on dual-species biofilms observed in this study might also be a result of changes in the QS system. However, determining the specific molecular mechanisms of this action requires additional research.

Despite the results of our study, future studies are needed to investigate whether norspermidine controls dental plaque in an ecological way, with no or fewer side effects. A molecule that reacts dissimilarly with different microorganism may be able to regulate microflora in an ecological way, through inhibiting pathogens while promoting probiotic bacteria, either directly or indirectly. Furthermore, the combination of norspermidine and other biochemical agents may provide a novel strategy for biofilm control.

## 5. Conclusions

Since norspermidine could regulate dual-species biofilms composed of *S. mutans* and *S. sanguinis* in an ecological way, norspermidine may be a potent reagent to control dental biofilms and manage dental caries.

## Data Availability

The datasets generated and analyzed to support the study are available from the corresponding author on reasonable request.

## Supplementary Materials

Supplementary Materials Supplementary Appendix Figure 1: lactic acid at 0 mM, 0.5 mM, 1.0 mM, 1.5 mM, 2.0 mM, 2.5 mM, 3.0 mM, and 3.5 mM were used as substrates for reaction with lactate dehydrogenase, respectively, to generate the standard curve. Supplementary Appendix Figure 2: dextran at 0 *μ*g/mL, 10 *μ*g/mL, 20 *μ*g/mL, 30 *μ*g/mL, 40 *μ*g/mL, 60 *μ*g/mL, 80 *μ*g/mL, and 100 *μ*g/mL were used for reaction with anthrone, respectively, to generate the standard curve. Supplementary Appendix Table 1: oligonucleotide primers of *S. mutans* and *S. sanguinis* used in fluorescent in situ hybridization (FISH).

## References

[1] Marsh P. D. (2004). Dental plaque as a microbial biofilm. *Caries Research*.

[2] Pitts N. B., Zero D. T., Marsh P. D. (2017). Dental caries. *Nature Reviews Disease Primers*.

[3] Schwendicke F., Korte F., Dörfer C. E., Kneist S., Fawzy El-Sayed K., Paris S. (2017). Inhibition of *Streptococcus mutans* growth and biofilm formation by probiotics in vitro. *Caries Research*.

[4] Galvão L. C., Rosalen P. L., Rivera-Ramos I. (2016). Inactivation of the spxA1 or spxA2 gene of *Streptococcus mutans* decreases virulence in the rat caries model. *Molecular Oral Microbiology*.

[5] Wang Y., Wang X., Jiang W. (2018). Antimicrobial peptide GH12 suppresses cariogenic virulence factors of *Streptococcus mutans*. *Journal of Oral Microbiology*.

[6] Li M., Huang R., Zhou X., Zhang K., Zheng X., Gregory R. L. (2014). Effect of nicotine on dual-species biofilms of *Streptococcus mutans* and *Streptococcus sanguinis*. *FEMS Microbiology Letters*.

[7] Jens K., Justin M., Wenyuan S., Fengxia Q. (2005). Competition and coexistence between *Streptococcus mutans* and *Streptococcus sanguinis* in the dental biofilm. *Journal of Bacteriology*.

[8] Kreth J., Zhang Y., Herzberg M. C. (2008). Streptococcal antagonism in oral biofilms: *Streptococcus sanguini*s and *Streptococcus gordonii* interference with *Streptococcus mutans*. *Journal of Bacteriology*.

[9] Valdebenito B., Tullume-Vergara P. O., González W., Kreth J., Giacaman R. A. (2017). In silico analysis of the competition between *Streptococcus sanguinis* and *Streptococcus mutans* in the dental biofilm. *Molecular Oral Microbiology*.

[10] Jeongmi L., Vanessa S., Frantz D. E. (2009). An alternative polyamine biosynthetic pathway is widespread in bacteria and essential for biofilm formation in *Vibrio cholerae*. *Journal of Biological Chemistry*.

[11] Laura H., Sok Ho K., Yukari M. (2014). Norspermidine is not a self-produced trigger for biofilm disassembly. *Cell*.

[12] Ramón-Peréz M. L., Díaz-Cedillo F., Contreras-Rodríguez A. (2015). Different sensitivity levels to norspermidine on biofilm formation in clinical and commensal *Staphylococcus epidermidis* strains. *Microbial Pathogenesis*.

[13] Qu L., She P., Wang Y. (2016). Effects of norspermidine onPseudomonas aeruginosabiofilm formation and eradication. *Microbiologyopen*.

[14] Nesse L. L., Berg K., Vestby L. K. (2015). Effects of norspermidine and spermidine on biofilm formation by potentially pathogenic *Escherichia coli* and *Salmonella enterica* wild-type strains. *Applied and Environmental Microbiology*.

[15] Cardile A. P., Woodbury R. L., Sanchez C. J. (2016). Activity of norspermidine on bacterial biofilms of multidrug-resistant clinical isolates associated with persistent extremity wound infections. *Advances in Experimental Medicine and Biology*.

[16] Ou M., Ling J. (2017). Norspermidine changes the basic structure of *S. mutans* biofilm. *Molecular Medicine Reports*.

[17] Peeters E., Nelis H. J., Coenye T. (2008). Comparison of multiple methods for quantification of microbial biofilms grown in microtiter plates. *Journal of Microbiological Methods*.

[18] Zheng X., Zhang K., Zhou X. (2013). Involvement of gshAB in the interspecies competition within oral biofilm. *Journal of Dental Research*.

[19] Shang D., Liang H., Wei S., Yan X., Yang Q., Sun Y. (2014). Effects of antimicrobial peptide L-K6, a temporin-1CEb analog on oral pathogen growth, *Streptococcus mutans* biofilm formation, and anti-inflammatory activity. *Applied Microbiology and Biotechnology*.

[20] Wang S.-P., Ge Y., Zhou X.-D. (2016). Effect of anti-biofilm glass-ionomer cement on *Streptococcus mutans* biofilms. *International Journal of Oral Science*.

[21] Cheng L., Zhang K., Zhou C.-C., Weir M. D., Zhou X.-D., Xu H. H. K. (2016). One-year water-ageing of calcium phosphate composite containing nano-silver and quaternary ammonium to inhibit biofilms. *International Journal of Oral Science*.

[22] Liu S., Qiu W., Zhang K. (2017). Nicotine enhances interspecies relationship between *Streptococcus mutans* and *Candida albicans*. *BioMed Research International*.

[23] Liu C., Niu Y., Zhou X. (2015). *Streptococcus mutans* copes with heat stress by multiple transcriptional regulons modulating virulence and energy metabolism. *Scientific Reports*.

[24] Zhang K., Wang S., Zhou X. (2015). Effect of antibacterial dental adhesive on multispecies biofilms formation. *Journal of Dental Research*.

[25] Takahashi N., Nyvad B. (2011). The role of bacteria in the caries process: ecological perspectives. *Journal of Dental Research*.

[26] Philip N., Suneja B., Walsh L. J. (2018). Ecological approaches to dental caries prevention: paradigm shift or shibboleth?. *Caries Research*.

[27] Koo H., Falsetta M. L., Klein M. I. (2013). The exopolysaccharide matrix: a virulence determinant of cariogenic biofilm. *Journal of Dental Research*.

[28] Koo H., Xiao J., Klein M. I. (2009). Extracellular polysaccharides matrix - an often forgotten virulence factor in oral biofilm research. *International Journal of Oral Science*.

[29] Bowen W. H., Burne R. A., Wu H., Koo H. (2018). Oral biofilms: pathogens, matrix, and polymicrobial interactions in microenvironments. *Trends in Microbiology*.

[30] Yasuo Y., Hiroyasu K., Keiji N. (2015). The influence of a glucosyltransferase, encoded by gtfP, on biofilm formation by *Streptococcus sanguinis* in a dual-species model. *APMIS*.

[31] Leme A. F. P., Koo H., Bellato C. M., Bedi G., Cury J. A. (2006). The role of sucrose in cariogenic dental biofilm formation-new insight. *Journal of Dental Research*.

[32] Lozano C. P., Díaz-Garrido N., Kreth J., Giacaman R. A. (2019). *Streptococcus mutans* and *Streptococcus sanguinis* expression of competition-related genes, under sucrose. *Caries Research*.

[33] Anderson C., Curzon C M. L., Tatsi C., Duggal M. (2010). Sucrose and dental caries: a review of the evidence. *Obesity Reviews*.

[34] Dashper S. G., Reynolds E. C. (2000). Effects of organic acid anions on growth, glycolysis, and intracellular pH of oral streptococci. *Journal of Dental Research*.

[35] Iwami Y., Yamada T. (1980). Rate-limiting steps of the glycolytic pathway in the oral bacteria *Streptococcus mutans* and *Streptococcus sanguis* and the influence of acidic pH on the glucose metabolism. *Archives of Oral Biology*.

[36] Si X., Quan X., Li Q., Wu Y. (2014). Effects of d-amino acids and norspermidine on the disassembly of large, old-aged microbial aggregates. *Water Research*.

