# Supplementary material for: Effects of Norspermidine on Dual‐Species Biofilms Composed of Streptococcus mutans and Streptococcus sanguinis
Source: Biomed Res Int. 2019 Nov 3;2019:1950790. doi: 10.1155/2019/1950790 (PMC6874952; doi:10.1155/2019/1950790)
Supplement: Supplementary Materials — Supplementary Appendix Figure 1: lactic acid at 0 mM, 0.5 mM, 1.0 mM, 1.5 mM, 2.0 mM, 2.5 mM, 3.0 mM, and 3.5 mM were used as substrates for reaction with lactate dehydrogenase, respectively, to generate the standard curve. Supplementary Appendix Figure 2: dextran at 0 μg/mL, 10 μg/mL, 20 μg/mL, 30 μg/mL, 40 μg/mL, 60 μg/mL, 80 μg/mL, and 100 μg/mL were used for reaction with anthrone, respectively, to generate the standard curve. Supplementary Appendix Table 1: oligonucleotide primers of S. mutans and S. sanguinis used in fluorescent in situ hybridization (FISH). [file 1950790.f1.docx]

Appendix Fig. 1 Standard curve generated by known concentration of lactic acid.

Lactic acid at 0 mM, 0.5 mM, 1.0 mM, 1.5mM, 2.0 mM, 2.5 mM. 3.0 mM and 3.5 mM were used as substrates for reaction with lactate dehydrogenase respectively to generate the standard curve.

Appendix Fig. 2 Standard curve generated by known concentration of dextran.

Dextran at 0μg/mL, 10μg/mL, 20μg/mL, 30μg/mL, 40μg/mL, 60μg/mL, 80μg/mL, 100μg/mL were used for reaction with anthrone respectively to generate the standard curve.

Appendix Table 1. Oligonucleotide primers of *S. mutans and S. sanguinis* used in fluorescent in situ hybridization (FISH).

| Probes | Nucelotide Sequence (5’-3’) | Reference |
| --- | --- | --- |
| *S. mutans*  *S.sanguinis* | Alexa Fluor 488-5’-ACTCCAGACTTTCCTGAC-3’  Alex Fluor 594-5’-GCATACTATGGTTAAGCCAC  AGCC-3’ | [[1](#_ENREF_1)]  [[1](#_ENREF_1)] |

**Appendix reference**

[1] Zheng X, Zhang K, Zhou X, Liu C, Li M, Li Y, et al. Involvement of gshAB in the interspecies competition within oral biofilm. Journal of Dental Research. 2013;92:819-24.
